# Association between Consumption of Coffee and the Prevalence of Periodontitis: The 2008–2010 Korea National Health and Nutrition Examination Survey

**DOI:** 10.1371/journal.pone.0158845

**Published:** 2016-07-07

**Authors:** Kyungdo Han, Eunkyung Hwang, Jun-Beom Park

**Affiliations:** 1 Department of Biostatistics, College of Medicine, The Catholic University of Korea, Seoul, Republic of Korea; 2 Bangmok College of General Education, Myongji University, Seoul, Republic of Korea; 3 Department of Periodontics, College of Medicine, The Catholic University of Korea, Seoul, Republic of Korea; University of Insubria, ITALY

## Abstract

**Background:**

This study was performed to assess the relationship between the consumption of coffee and periodontitis using nationally representative data.

**Methods:**

The data from the Korea National Health and Nutrition Examination Survey were used; the analysis in this study was confined to a total of 16,730 respondents over 19 years old who had no missing values for the consumption of coffee or outcome variables. A community periodontal index greater than or equal to code 3 was defined as periodontal disease.

**Results:**

Consumption of coffee was significantly higher in the individuals with periodontitis in males. The odds ratios of the percentage of individuals with periodontitis tended to increase with the consumption of coffee. Adjusted odds ratios and their 95% confidence intervals of the male participants were 1, 1.131(0.792–1.617), 1.161(0.857–1.573), 1.053(0.805–1.379), 1.299(1.007–1.676), and 1.458(1.141–1.862) for once per month or less, once per month<x≤3 times per week, three times per week<x≤6 times per week, once per day, twice per day, and three or more per day, respectively.

**Conclusions:**

Consumption of coffee may be considered an independent risk indicator of periodontal disease in Korean male adults, and we suggest that the periodontal health of male may benefit from reduction of coffee consumption.

## Introduction

Coffee is one of the most frequently consumed caffeine-containing beverages[1,2]. Coffee has been suggested to be associated with many health conditions, and coffee consumption has been historically linked to adversehealth effects[1]. Epidemiological studies suggested that consumption of boiled coffee was associated with elevated risk for cardiovascular disease[3]. Consumption of coffee, particularly instant coffee mix, may have harmful effects on metabolic syndrome[4]. Specific dietary patterns, which include coffee patterns, were reported to be independently associated with obesity[5]. However, the results of epidemiological research suggested that coffee consumption may help prevent several chronic diseases. Coffee intake was inversely associated with type 2 diabetes[6,7], and coffee consumption was associated with decreased insulin resistance[8]. Coffee drinking probably confers protection against alcohol-related increase in liver enzymes[9], and coffee consumption reduced the risk of hepatocellular carcinoma and colon ademomas[10,11].

One report showed a negative influence on bone metabolism, but another report demonstrated that coffee consumption showed no significant association with bone mineral density[12,13]. Periodontitis is a chronic inflammatory disease of periodontal tissue[14,15], and it is not only a local phenomenon but is also related with systemic conditions[16]. Thus, this study was performed to assess the relationship between the consumption of coffee and periodontitis using nationally representative data.

## Materials and Methods

### Overview of the survey and participants

The data used in this study were derived from the Korea National Health and Nutrition Examination Survey (KNHANES), conducted between 2008 and 2010 by the Division of Chronic Disease Surveillance under the Korea Centers for Disease Control and Prevention and the Korean Ministry of Health and Welfare. The KNHANES is a nationwide survey of noninstitutionalized civilians who uses a stratified and multistage probability sampling design with a rolling survey sampling model[17]. The sampling units were based on the population and housing consensus from the 2005 National Census Registry in Korea, which includes age, gender, and geographic area. The sample weights were used to calculate all statistics of this survey. To represent the Korean population with sample participants, sample weights were created, considering survey nonresponse, complex survey design, and post-stratification[3].

Initially, a total of 29,235 individuals participated in the KNHANES survey. The 7,424 subjects aged < 30 were excluded, and the 3,681 participants without nutritional information were excluded. Among the remaining 17,058 patients, 1,072 subjects who had periodontitis with a community periodontal index (CPI) of 3 were selected. After removing 328 subjects who had missing parameters, 16,730 total subjects were included in this study. All of the participants received written informed consent. This study was approved by the Institutional Review Board of the Korean Center for Disease Control and Prevention and conducted according to the Helsinki Declaration-based Ethical Principles for Medical Research Involving Human Subjects. The Institutional Review Board at the Catholic University of Korea approved this study.

### Sociodemographic and lifestyle variables

All participants were asked about sociodemographic and lifestyle variables by trained interviewers. Consumption of coffee and carbonated beverages was calculated based on the survey. Education level was categorized into two groups according to whether or not the participant had graduated from high school. Monthly household income was divided into quartiles after adjusting for the number of family members. The lowest quartile included households with a monthly income < $1092.40 USD. The amount of pure alcohol consumed (in grams per day) was calculated using the average number of alcoholic beverages consumed and the frequency of alcohol consumption. The participants were divided into three groups depending on the amount of alcohol consumed per day: nondrinker, light-to-moderate drinker (1–30 g/day), and heavy drinker (> 30 g/day)[18]. Smoking status was categorized into three groups according to respondents’ answers on the self-report questionnaire: nonsmokers, ex-smokers, and current smokers[14]. Based on responses to the modified form of the International Physical Activity Questionnaire for Koreans, individuals were regarded as regular physical exercisers if they performed moderate exercise more than five times per week for at least 30 minutes per session or performed vigorous exercise more than three times per week for at least 20 minutes per session[19]. Daily energy, fat, protein, and calcium intake were calculated based on the survey.

### Anthropometric measurements

Anthropometric measurements were performed by trained staff members. Body weight and height were measured to the nearest 0.1 kg and 0.1 cm, respectively, with participants in light indoor clothing without shoes. Body mass index was calculated as body weight (kg) divided by the squared height (m^2^), and waist circumference was measured at the narrowest point between the lower border of the ribcage and the iliac crest in a standing position.

### Biochemical measurements

The physical measurements of the participants were taken by trained staff members of the Division of Chronic Disease Surveillance under the Korea Centers for Disease Control and Prevention and the Korean Ministry of Health and Welfare.

A standard mercury sphygmomanometer (Baumanometer, W. A. Baum Co., Copiague, NY, USA) was used for blood pressure measurement. Systolic blood pressure and diastolic blood pressure were measured twice at five-minute intervals, and the average values were used for the analysis.

To measure concentrations of serum-fasting plasma glucose and high-density lipoprotein cholesterol and white blood cell count, a blood sample was collected from the antecubital vein of each participant after fasting for more than eight hours. Blood samples were analyzed within 24 hours of transportation. Levels of serum-fasting plasma glucose, total cholesterol, triglycerides, and high-density lipoprotein cholesterol were measured with a Hitachi Automatic Analyzer 7600 (Hitachi, Tokyo, Japan) by enzymatic methods using commercially available kits (Daiichi, Tokyo, Japan)[20].

### Description of metabolic syndrome and diabetes

Metabolic syndrome was defined according to the American Heart Association/National Heart, Lung, and Blood Institute Scientific Statement criteria for Asians[21]. According to these criteria, three or more of the following criteria must be fulfilled to be diagnosed with metabolic syndrome: waist circumference ≥ 90 cm for men and ≥ 80 cm for women; fasting triglycerides ≥ 150 mg/dL or use of lipid-lowering medication; high-density lipoprotein cholesterol < 40 mg/dL in men and < 50 mg/dL in women or use of medication; blood pressure ≥ 130/85 mmHg or use of hypertension medication; and fasting blood glucose ≥ 100 mg/dL or current use of diabetes medication[22]. Diabetes is defined as fasting blood sugar greater than 126 mg/dL or when the individual was currently using anti-diabetic medications[23].

### Periodontal treatment needs and oral health behaviors

Tooth brushing frequency and the use of secondary oral products were used as indicators of oral health behavior. The frequency of daily tooth brushing was calculated as the total number of times the teeth were brushed per day. Secondary oral products included dental floss, mouthwash, interdental brush, and electric toothbrush[24].

The World Health Organization community periodontal index (CPI) was used to assess periodontal treatment needs and defined periodontal disease as a CPI greater than or equal to code 3. A CPI score of code 3 indicates that at least one site had a > 3.5 mm pocket in the index teeth, which are 11, 16, 17, 26, 27, 31, 36, 37, 46, and 47 according to the Federation DentaireInternationale system. The mouth was divided into sextants. A CPI probe (PWHO, Osung MND, Seoul, South Korea) with a 0.5 mm ball tip was used. A sextant was examined only if there were two or more teeth present that were not scheduled for extraction. If no index teeth were present in a sextant qualifying for examination, all remaining teeth were examined and the highest score was recorded as the score for that sextant. An approximate 20-gram probing force was used. Trained and calibrated dentists examined the periodontal status of the participants. Chewing ability, speech, dental checkup within a year, and self-reported oral status were evaluated. The CPI score represented periodontitis status as follows: 0: normal, 1: gingival bleeding, 2: calculus, 3: a shallow pocket with depth of 3.5–5.5 mm or moderate periodontitis, and 4: a deep pocket depth ≥ 5.5 mm or severe periodontitis[25]. Dental checkup within the past year, self-reported oral status, chewing ability and speech, and were evaluated.

### Statistical analyses

All data are presented as mean ± standard error or as a percentage (standard error). Logarithmic transformation was performed to achieve normal distribution when necessary. A student’s t-test or chi-square test was used to investigate the differences in the presence of periodontal treatment needs according to the variables. Multiple logistic regression analyses were used to assess the associations of periodontal treatment needs and consumption of carbonated beverages. The model was adjusted for age, sex, body mass index, smoking, drinking, exercise, metabolic syndrome, frequency of tooth brushing per day, use of secondary oral products, dental examination within a year, and consumption of beer and coffee. A survey procedure of a statistical software package (SAS version 9.2 for Windows, SAS Institute, Cary, NC, USA) was used for statistical analysis to account for the complex sampling design. Two-sided *P* values of < 0.05 were considered statistically significant.

## Results

Table 1 describes the baseline characteristics of the study individuals according to the presence of periodontal treatment needs. The mean age, body mass index, and waist circumference were significantly higher in participants with periodontal diseasein both male and female. Consumption of coffee and consumption of carbonated beverage were significantly higher in the individuals with periodontitis in male. Use of secondary oral products was significantly lower in participants with periodontal disease.

**Table 1:** Baseline characteristics of study individuals according to periodontal disease.

	Periodontitis					
	No	Yes	*P* value*	No	Yes	*P* value*
	Male			Female		
Unweighted n	3971	2745		7321	2693	
Age (years)	39.15±0.32	50.42±0.36	<0.0001	41.77±0.29	54.51±0.37	<0.0001
Weight (kg)	70.88±0.22	68.95±0.26	<0.0001	57.07±0.13	58.23±0.23	<0.0001
Height (m)	1.715±0.001	1.69±0.002	<0.0001	1.581±0.001	1.552±0.002	<0.0001
Body mass index (kg/m^2^)	24.05±0.06	24.09±0.08	0.6839	22.85±0.06	24.19±0.1	<0.0001
Waist circumference (cm)	83.53±0.2	85±0.21	<0.0001	76.64±0.18	81.53±0.31	<0.0001
**Consumption of coffee**			<0.0001			0.1485
≤ Once per month	16.7(0.7)	11.2(0.7)		18.3(0.6)	19.5(0.9)	
Once per month<x≤3 times per week	7.4(0.5)	5.5(0.5)		8(0.4)	7.6(0.6)	
Three times per week<x≤6 times per week	12.5(0.6)	10.4(0.7)		13.4(0.5)	11.1(0.7)	
Once per day	20.8(0.8)	20.1(0.9)		27.3(0.6)	28.8(1.1)	
Twice per day	18.7(0.7)	23.3(1)		20.5(0.6)	20.3(1)	
Three or more per day	23.9(0.8)	29.5(1.2)		12.4(0.5)	12.6(0.8)	
**Consumption of carbonated beverage**			<0.0001			-
≤ Once per month	44.9(1)	62.4(1.2)		66(0.8)	78.2(1)	
Once per month<x≤3 times per week	28.6(0.8)	23.1(1)		22.8(0.7)	16.2(0.8)	
Three times per week<x≤6 times per week	22.3(0.9)	12.9(0.9)		9.6(0.5)	5.0(0.5)	
Once per day	3.7(0.4)	1.4(0.3)		1.3(0.2)	0.5(0.2)	
Twice per day	0.5(0.1)	0.1(0.1)		0.2(0.1)	-	
Three or more per day	0(0)	0.1(0.1)		0.1(0.0)	-	
Energy intake (kcal/day)	2401.3±19.2	2285.3±23	<0.0001	1689.9±10	1576.6±15.6	<0.0001
Percentage fat in total energy intake (%)	20.12±0.18	17.39±0.22	<0.0001	17.9±0.14	14.38±0.19	<0.0001
Percentage protein in total energy intake (%)	15.54±0.09	15.07±0.1	<0.0001	14.35±0.07	13.74±0.09	<0.0001
Calcium intake (mg/day)	576.6±6.5	570.6±9.1	0.0006	460.1±4.9	441.3±9.7	<0.0001
**Smoking**			<0.0001			0.0756
Nonsmoker	27.2(0.8)	15.8(0.8)		89.4(0.5)	89.7(0.8)	
Ex-smoker	27.7(0.8)	35.8(1)		4.8(0.3)	3.6(0.4)	
Current smoker	45.1(0.9)	48.4(1.2)		5.8(0.4)	6.7(0.7)	
**Drinking**			<0.0001			<0.0001
Nondrinker	11.2(0.6)	17.2(0.8)		30.7(0.7)	41.4(1.2)	
Light-to-moderate drinker	71.2(0.8)	63.4(1.1)		66.9(0.7)	57(1.2)	
Heavy drinker	17.6(0.7)	19.5(1)		2.3(0.3)	1.7(0.3)	
Education (high school graduate or higher)	84.6(0.7)	65.2(1.2)	<0.0001	73.2(0.7)	42(1.5)	<0.0001
Income (the lowest quartile)	12.7(0.8)	17.0(0.9)	0.0001	14.3(0.6)	24.1(1.1)	<0.0001
Exercise (yes)	28(0.9)	28.2(1.1)	0.9258	22.2(0.7)	24.5(1.1)	0.0548
Residential place (rural)	83.1(1.6)	76.2(2.1)	<0.0001	83.1(1.5)	76.3(2.2)	<0.0001
Occupation (yes)	76.2(0.9)	79.4(1)	0.0128	49.1(0.8)	46.1(1.4)	0.0464
White blood cell (x10^3^/μL) **	6.3(6.24–6.36)	6.57(6.5–6.64)	0.5925	5.53(5.48–5.57)	5.7(5.63–5.77)	0.0817
Metabolic syndrome	23.2(0.8)	37.5(1.2)	<0.0001	17.6(0.6)	38.7(1.2)	<0.0001
Diabetes	5.4(0.4)	14.6(0.8)	<0.0001	5.5(0.3)	12.5(0.8)	<0.0001
Frequency of tooth brushing per day			<0.0001			<0.0001
≤ 1	13.4(0.7)	18.9(0.9)		7.3(0.4)	11.3(0.9)	
2	44.4(1)	45.9(1.2)		40.7(0.7)	47.4(1.1)	
≥ 3	42.3(1)	35.1(1.2)		52(0.8)	41.4(1.2)	
Use of secondary oral products (yes)	38.6(1.1)	34.1(1.4)	0.0129	48.6(0.9)	31.3(1.2)	<0.0001
Dental checkup within a year (yes)	27.3(1)	27.5(1.2)	0.8767	27.8(0.8)	22.7(1.1)	<0.0001
**Self-reported oral status**			<0.0001			<0.0001
Favorable	15.6(0.6)	9.6(0.6)		12.3(0.5)	7.7(0.7)	
Average	45(0.9)	31.2(1.2)		44.3(0.8)	32.4(1.2)	
Problematic	39.4(0.9)	59.3(1.3)		43.4(0.8)	59.9(1.2)	
**Chewing**			<0.0001			<0.0001
Discomfort	19.3(0.8)	39.2(1.2)		21.9(0.6)	43.8(1.1)	
Minor problem	15.4(0.8)	16.3(0.9)		15.2(0.6)	14.5(0.8)	
No discomfort	65.2(1)	44.6(1.2)		62.9(0.8)	41.7(1.1)	
**Speech**			<0.0001			<0.0001
Discomfort	5(0.4)	10.6(0.7)		6.3(0.3)	14.2(0.8)	
Minor problem	5.8(0.4)	9.8(0.7)		6.7(0.4)	9.9(0.7)	
No discomfort	89.2(0.6)	79.7(1)		87(0.5)	75.9(1)	

Data are presented as mean ± standard error or percentages (standard error).

* *P* values were obtained by independent t-test for continuous variables or chi-square test for categorical variables.

** Log transformation was applied to the value, and geometric mean (95% confidence of interval) was shown.

Table 2 shows the subgroup analysis regarding the consumption of coffee categorized by sex. Self-reported oral status is related with the consumption of coffee in males. Statistically significant association is seen regarding chewing and speech with consumption of coffee in both males and females.

**Table 2:** Subgroup analysis regarding the consumption of coffee categorized by sex.

	Male							Female						
	Consumption of coffee													
	≤ Once per month	Once per month<x≤3 times per week	Three times per week<x≤6 times per week	Once per day	Twice per day	Three or more per day	*P* value	≤ Once per month	Once per month<x≤3 times per week	Three times per week<x≤6 times per week	Once per day	Twice per day	Three or more per day	*P* value
**Frequency of tooth brushing per day**							0.2043							0.2043
≤ 1	16.2(1.5)	15.1(2.1)	14.81.6)	17.3(1.1)	14.0(1.1)	14.5(1.0)		10.9(0.9)	11.8(1.5)	6.8(0.9)	7.4(0.7)	7.1(0.7)	6.9(0.9)	
2	42.4(2.0)	44.8(3.2)	45.1(2.3)	42.9(1.7)	44.2(1.6)	48.6(1.5)		42.8(1.4)	43.4(2.0)	42.7(1.7)	43.4(1.2)	41.3(1.4)	39.6(1.7)	
≥ 3	41.4(2.0)	40.1(3.0)	40.1(2.3)	39.8(1.7)	41.8(1.6)	36.9(1.4)		46.4(1.4)	44.7(2.0)	50.5(1.7)	49.2(1.2)	51.6(1.5)	53.5(1.7)	
**Use of secondary oral products**							0.2427							0.0008
No	65.7(2.1)	62.1(3.5)	60.0(2.4)	65.8(1.7)	61.7(1.8)	61.9(1.6)		60.6(1.5)	59.4(2.4)	54.1(2.0)	54.8(1.3)	53.9(1.5)	51.4(1.9)	
Yes	34.3(2.1)	37.9(3.5)	40.0(2.4)	34.2(1.7)	38.3(1.8)	38.1(1.6)		39.4(1.5)	40.6(2.4)	45.9(2.0)	45.2(1.3)	46.1(1.5)	48.6(1.9)	
**Dental checkup within a year**							0.1574							0.0989
No	74.0(1.8)	75.2(2.5)	75.0(1.9)	73.7(1.5)	70.4(1.6)	71.0(1.4)		76.4(1.2)	72.7(2.0)	71.2(1.6)	72.6(1.2)	72.8(1.3)	74.3(1.7)	
Yes	26.0(1.8)	24.8(2.5)	25.0(1.9)	26.3(1.5)	29.6(1.6)	29.0(1.4)		23.6(1.2)	27.3(2)	28.8(1.6)	27.4(1.2)	27.2(1.3)	25.7(1.7)	
**Self-reported oral status**							0.0057							0.0193
Favorable	15.2(1.3)	15.4(2)	16.2(1.6)	13.1(1)	14.1(1.1)	10.7(0.9)		10.6(0.9)	14.5(1.7)	12.7(1.1)	11.3(0.7)	10.0(0.8)	10.3(1.1)	
Average	41.5(1.8)	44.1(3.2)	39.8(2.2)	40.5(1.6)	40.0(1.6)	38.3(1.5)		41.0(1.3)	39.1(2.1)	45.0(1.8)	41.9(1.1)	41.6(1.4)	39.3(1.8)	
Problematic	43.3(1.9)	40.5(2.9)	44.0(2.3)	46.5(1.7)	45.8(1.6)	51.0(1.6)		48.5(1.4)	46.4(2.3)	42.4(1.7)	46.8(1.2)	48.4(1.4)	50.4(1.8)	
**Chewing**							0.0015							<0.0001
Discomfort	22.3(1.5)	24.1(2.6)	24.0(1.8)	27.8(1.5)	24.5(1.4)	30.3(1.4)		30.4(1.2)	29.4(2.1)	24.6(1.3)	27.3(1.0)	24.8(1.1)	26.4(1.4)	
Minor problem	14.6(1.3)	14.6(2.3)	14.5(1.6)	17.4(1.3)	17.3(1.2)	14.6(1.1)		14.3(0.9)	12.6(1.4)	13.1(1.1)	17.1(0.9)	13.9(0.9)	16.7(1.3)	
No discomfort	63.1(1.8)	61.3(3.0)	61.5(2.1)	54.8(1.7)	58.1(1.6)	55.1(1.5)		55.3(1.4)	58.0(2.2)	62.3(1.6)	55.6(1.1)	61.3(1.3)	56.9(1.7)	
**Speech**							0.0023							0.0414
Discomfort	6.5(0.8)	9.5(1.5)	6.1(1.0)	6.8(0.8)	6.7(0.7)	7.3(0.7)		9.8(0.8)	9.6(1.3)	7.4(0.8)	8.2(0.6)	6.8(0.6)	7.8(0.9)	
Minor problem	6.4(0.9)	4.7(1.1)	5.5(0.9)	10.0(1.0)	6.3(0.7)	7.6(0.8)		8.8(0.8)	7.9(1.2)	6.6(0.7)	6.9(0.6)	7.2(0.7)	7.4(0.9)	
No discomfort	87.2(1.2)	85.9(1.8)	88.4(1.2)	83.3(1.2)	87.1(1.0)	85.1(1.1)		81.4(1.0)	82.5(1.7)	86.0(1.0)	84.8(0.8)	86.0(0.9)	84.9(1.2)	

Data are presented as percentages (standard error).

Table 3 shows the adjusted odds ratios of periodontitis using the multivariate logistic regression model for consumption of coffee. Adjusted odds ratios and their 95% confidence intervals of the male participants were 1, 1.131(0.792–1.617), 1.161(0.857–1.573), 1.053(0.805–1.379), 1.299(1.007–1.676), and 1.458(1.141–1.862) for once per month or less, once per month<x≤3 times per week, three times per week<x≤6 times per week, once per day, twice per day, and three or more per day, respectively (*P* for trend < 0.05). Adjusted odds ratios and their 95% confidence intervals of the female participants were 1, 1.154(0.862–1.545), 0.951(0.750–1.205), 0.941(0.771–1.148), 1.034(0.825–1.296), and 1.265(0.969–1.65) for once per month or less, once per month<x≤3 times per week, three times per week<x≤6 times per week, once per day, twice per day, and three or more per day, respectively (*P* for trend > 0.05). The results show that consumption of coffee was positively associated with the risk of periodontal disease in Korean male adults.

**Table 3:** Adjusted odds ratio, 95% confidence interval and *P* value of prevalence of periodontitis in multivariate logistic regression model for consumption of coffee.

Consumption of coffee	Male	Female
≤ Once per month	1	1
Once per month<x≤3 times per week	1.131(0.792–1.617)	1.154(0.862–1.545)
Three times per week<x≤6 times per week	1.161(0.857–1.573)	0.951(0.750–1.205)
Once per day	1.053(0.805–1.379)	0.941(0.771–1.148)
Twice per day	1.299(1.007–1.676)	1.034(0.825–1.296)
Three or more per day	1.458(1.141–1.862)	1.265(0.969–1.65)
*P* for trend	0.0013	0.3454

Model: age, body mass index, smoking, drinking, exercise, metabolic syndrome, frequency of tooth brushing per day, use of secondary oral products, consumption of carbonated beverage, and dental checkup within a yearadjusted.

## Discussion

In the present study, the risk of periodontal disease was positively associated with consumption of coffee among Korean male adults. This association between consumption of carbonated beverages and periodontal disease was independent of various potential confounding factors such as age, body mass index, smoking, drinking, exercise, metabolic syndrome, frequency of tooth brushing per day, use of secondary oral products, consumption of carbonated beverage, and dental checkup within a year.

Limited numbers of studies have been performed regarding the effect of coffee consumption on periodontal disease[26,27,28,29,30,31,32] Previous study showed that heavy coffee consumption is associated with a significant increase in risk of fracture, osteoporosis, and periodontal disease[26]. The association between periodontitis and the common risk factors of coffee was confirmed in the previous research[27]. Another study showed that coffee consumption was independently associated with an increased prevalence of tooth loss[28]. The daily ingestion of coffee is shown to delay the alveolar bone reparative process after tooth extraction[29]. Conversely, some suggested that coffee consumption has no deleterious effects on periodontal health[30], and coffee did not stimulate bone loss in rats[26]. It was suggested that coffee consumption may be protective against periodontal bone loss in adult males[31]. An inverse association between coffee consumption (≥1 cup/day) and prevalence of severe periodontitis was found in the maintenance phase of periodontal treatment[32].

The different results for the association between the consumption of coffee and periodontitis may be partially explained by the following. Coffee contains complex chemicals, including carbohydrates, nitrogenous compounds, vitamins, minerals, and phenolic compounds[1,2]. Coffee is considered to contain a dietary source of antioxidants as well as of other anti-inflammatory factors[31]. Coffee is also known to contain several potential anticarcinogenic and chemopreventive compounds[10]. Unfiltered coffee is a significant source of cafestol and kahweol, which are diterpenes that have been implicated in the cholesterol-raising effects of coffee[33]. Coffee contains caffeine, which is the most widely consumed psychoactive substance in the world[34]. In the general population, the main contributors to the total caffeine intake were coffee for adults, and a cup of coffee is usually regarded to contain 70 to 100 mg/cup[33,35,36]. Caffeine is reported to exert multiple effects on bone metabolism[37], and chronic caffeine intake is one of the possible risk factors in the advancement of pathology in the periodontitis patient[38]. Caffeine is shown to increase bone loss and reduce bone healing after tooth extraction[12].

A previous report showed that 76% of the Korean subjects were habitual coffee drinkers, most of whom consumed instant coffee mix containing sugar and powder creamer[4]. Drinking coffee with sugar can be tooth-damaging[39], and the worst tooth loss occurred among the women who drank coffee sweetened with sugar or syrup[40]. The groups using sugar, other sweetening agents, or neither of these, mainly in coffee, differed significantly and the nonusers of sugar had lowest periodontal treatment time[41]. The previous report also showed that consumption of coffee, particularly instant coffee mix, may result in weight gain and insulin resistance and may have harmful effects on metabolic syndrome, perhaps partly deriving from excessive intake of sugar and powder creamer[4,42]. The Kaplan-Meier survival analysis revealed that the risk of diabetes progression was lowest in patients who drank black coffee three or more times per day[43]. The instant coffee mix that contains nondairy creamer and/or sugar may have offset the potential benefit of coffee[42].

The major strength of our study is the large, nationally representative sample of adult Koreans[44]. Second, the natural teeth numbers and oral condition were objectively assessed by dentists using the same criteria, and several confounding variables were precisely recorded by well-trained examiners[45]. However, there are some limitations in the design of this study. This study is cross-sectional, and the exposure and outcome are measured at the same time, making their interrelated sequences unknown[14]. This study uses partial-mouth recording protocols of CPI, and partial-mouth recording protocols may underestimate the prevalence of periodontal disease[18]. The information regarding the coffee consumption was derived from the survey with frequency questionnaires and disparities that may exist between data details and the actual history[42].

The results showed that consumption of coffee was positively associated with the risk of periodontal disease in Korean male adults. In conclusion, consumption of coffee may be considered an independent risk indicator of periodontal disease in Korean male adults, and we suggest that the periodontal health of male individuals may benefit from the reduction of coffee consumption.

## Supporting Information

S1 Table STROBE Statement. (DOC)

## References

[1] Gonzalez de Mejia E, Ramirez-Mares MV (2014) Impact of caffeine and coffee on our health. Trends Endocrinol Metab 25: 489–492. 10.1016/j.tem.2014.07.003 25124982

[2] Kim SY (2014) Coffee consumption and risk of osteoporosis. Korean J Fam Med 35: 1 10.4082/kjfm.2014.35.1.1 24501663 PMC3912260

[3] Han K, Ko Y, Park YG, Park JB (2016) Associations Between the Periodontal Disease in Women Before Menopause and Menstrual Cycle Irregularity: The 2010–2012 Korea National Health and Nutrition Examination Survey. Medicine (Baltimore) 95: e2791. 26871840 10.1097/MD.0000000000002791 PMC4753936

[4] Kim HJ, Cho S, Jacobs DR Jr., Park K (2014) Instant coffee consumption may be associated with higher risk of metabolic syndrome in Korean adults. Diabetes Res Clin Pract 106: 145–153. 10.1016/j.diabres.2014.07.007 25112922

[5] Kim J, Jo I, Joung H (2012) A rice-based traditional dietary pattern is associated with obesity in Korean adults. J Acad Nutr Diet 112: 246–253. 10.1016/j.jada.2011.10.005 22732459

[6] Lee JK, Kim K, Ahn Y, Yang M, Lee JE (2015) Habitual coffee intake, genetic polymorphisms, and type 2 diabetes. Eur J Endocrinol 172: 595–601. 10.1530/EJE-14-0805 25755232

[7] Lin WY, Xaiver Pi-Sunyer F, Chen CC, Davidson LE, Liu CS, et al (2011) Coffee consumption is inversely associated with type 2 diabetes in Chinese. Eur J Clin Invest 41: 659–666. 10.1111/j.1365-2362.2010.02455.x 21226707 PMC3087821

[8] Pham NM, Nanri A, Kochi T, Kuwahara K, Tsuruoka H, et al (2014) Coffee and green tea consumption is associated with insulin resistance in Japanese adults. Metabolism 63: 400–408. 10.1016/j.metabol.2013.11.008 24342074

[9] Ikeda M, Maki T, Yin G, Kawate H, Adachi M, et al (2010) Relation of coffee consumption and serum liver enzymes in Japanese men and women with reference to effect modification of alcohol use and body mass index. Scand J Clin Lab Invest 70: 171–179. 10.3109/00365511003650165 20205615

[10] Budhathoki S, Iwasaki M, Yamaji T, Sasazuki S, Tsugane S (2015) Coffee intake and the risk of colorectal adenoma: The colorectal adenoma study in Tokyo. Int J Cancer 137: 463–470. 10.1002/ijc.29390 25500898

[11] Setiawan VW, Wilkens LR, Lu SC, Hernandez BY, Le Marchand L, et al (2015) Association of coffee intake with reduced incidence of liver cancer and death from chronic liver disease in the US multiethnic cohort. Gastroenterology 148: 118–125; quiz e115. 10.1053/j.gastro.2014.10.005 25305507 PMC4274222

[12] Bezerra JP, da Silva LR, de Alvarenga Lemos VA, Duarte PM, Bastos MF (2008) Administration of high doses of caffeine increases alveolar bone loss in ligature-induced periodontitis in rats. J Periodontol 79: 2356–2360. 10.1902/jop.2008.080204 19053927

[13] Choi EJ, Kim KH, Koh YJ, Lee JS, Lee DR, et al (2014) Coffee consumption and bone mineral density in korean premenopausal women. Korean J Fam Med 35: 11–18. 10.4082/kjfm.2014.35.1.11 24501665 PMC3912261

[14] Choi HM, Han K, Park YG, Park JB (2015) Associations Among Oral Hygiene Behavior and Hypertension Prevalence and Control: The 2008 to 2010 Korea National Health and Nutrition Examination Survey. J Periodontol 86: 866–873. 10.1902/jop.2015.150025 25741579

[15] Eke PI, Dye BA, Wei L, Thornton-Evans GO, Genco RJ (2012) Prevalence of periodontitis in adults in the United States: 2009 and 2010. J Dent Res 91: 914–920. 22935673 10.1177/0022034512457373

[16] Han K, Nam GE, Kim do H, Park JB, Ko Y, et al (2015) Association of Periodontitis With Urinary Albumin Excretion in Korean Adults With Diabetes: The 2012 Korea National Health and Nutrition Examination Survey. Medicine (Baltimore) 94: e1839. 26496329 10.1097/MD.0000000000001839 PMC4620835

[17] Han K, Ko Y, Park YG, Park JB (2016) Associations between the number of natural teeth in postmenopausal women and duration of lactation: The 2010–2012 Korea National Health and Nutrition Examination Survey. Maturitas 85: 73–78. 10.1016/j.maturitas.2015.12.010 26857883

[18] Park JB, Han K, Park YG, Ko Y (2014) Association between alcohol consumption and periodontal disease: the 2008 to 2010 Korea National Health and Nutrition Examination Survey. J Periodontol 85: 1521–1528. 10.1902/jop.2014.130782 25008215

[19] Oh JY, Yang YJ, Kim BS, Kang JH (2007) Validity and reliability of Korean version of International Physical Activity Questionnaire (IPAQ) short form. Journal of the Korean Academy of Family Medicine 28: 532–541.

[20] Wallace TM, Levy JC, Matthews DR (2004) Use and abuse of HOMA modeling. Diabetes Care 27: 1487–1495. 15161807 10.2337/diacare.27.6.1487

[21] Chun YH, Kim HR, Han K, Park YG, Song HJ, et al (2013) Total cholesterol and lipoprotein composition are associated with dry eye disease in Korean women. Lipids Health Dis 12: 84 10.1186/1476-511X-12-84 23734839 PMC3680171

[22] Kim YH, Cho KH, Choi YS, Kim SM, Nam GE, et al (2013) Low bone mineral density is associated with metabolic syndrome in South Korean men but not in women: The 2008–2010 Korean National Health and Nutrition Examination Survey. Arch Osteoporos 8: 142 10.1007/s11657-013-0142-3 23715738

[23] Jeon JY, Ko SH, Kwon HS, Kim NH, Kim JH, et al (2013) Prevalence of Diabetes and Prediabetes according to Fasting Plasma Glucose and HbA1c. Diabetes Metab J 37: 349–357. 10.4093/dmj.2013.37.5.349 24199164 PMC3816136

[24] Kim YH, Kim DH, Lim KS, Ko BJ, Han BD, et al (2013) Oral health behaviors and metabolic syndrome: the 2008–2010 Korean National Health and Nutrition Examination Survey. Clin Oral Investig. 10.1007/s00784-013-1112-2 24061606

[25] Islam SA, Seo M, Lee YS, Moon SS (2015) Association of periodontitis with insulin resistance, beta-cell function, and impaired fasting glucose before onset of diabetes. Endocr J 62: 981–989. 10.1507/endocrj.EJ15-0350 26329671

[26] Sakamoto W, Nishihira J, Fujie K, Iizuka T, Handa H, et al (2001) Effect of coffee consumption on bone metabolism. Bone 28: 332–336. 11248666 10.1016/s8756-3282(00)00444-0

[27] Zuccarello D, Bazzato MF, Ferlin A, Pengo M, Frigo AC, et al (2014) Role of familiarity versus interleukin-1 genes cluster polymorphisms in chronic periodontitis. Gene 535: 286–289. 10.1016/j.gene.2013.11.016 24275344

[28] Tanaka K, Miyake Y, Sasaki S, Ohya Y, Matsunaga I, et al (2008) Beverage consumption and the prevalence of tooth loss in pregnant Japanese women: the Osaka Maternal and Child Health Study. Fukuoka Igaku Zasshi 99: 80–89. 18646593

[29] Macedo RM, Brentegani LG, Lacerda SA (2015) Effects of coffee intake and intraperitoneal caffeine on bone repair process—a histologic and histometric study. Braz Dent J 26: 175–180. 10.1590/0103-6440201300219 25831110

[30] Duarte PM, Reis AF (2015) Coffee consumption has no deleterious effects on periodontal health but its benefits are uncertain. J Evid Based Dent Pract 15: 77–79. 10.1016/j.jebdp.2015.03.010 25987390

[31] Ng N, Kaye EK, Garcia RI (2014) Coffee consumption and periodontal disease in males. J Periodontol 85: 1042–1049. 10.1902/jop.2013.130179 24359164

[32] Machida T, Tomofuji T, Ekuni D, Azuma T, Takeuchi N, et al (2014) Severe periodontitis is inversely associated with coffee consumption in the maintenance phase of periodontal treatment. Nutrients 6: 4476–4490. 10.3390/nu6104476 25338270 PMC4210930

[33] Tan EK, Tan C, Fook-Chong SM, Lum SY, Chai A, et al (2003) Dose-dependent protective effect of coffee, tea, and smoking in Parkinson's disease: a study in ethnic Chinese. J Neurol Sci 216: 163–167. 14607318 10.1016/j.jns.2003.07.006

[34] Broderick P, Benjamin AB (2004) Caffeine and psychiatric symptoms: a review. J Okla State Med Assoc 97: 538–542. 15732884

[35] Lim HS, Hwang JY, Choi JC, Kim M (2015) Assessment of caffeine intake in the Korean population. Food Addit Contam Part A Chem Anal Control Expo Risk Assess 32: 1786–1798. 10.1080/19440049.2015.1077396 26248183

[36] Bae J, Park PS, Chun BY, Choi BY, Kim MK, et al (2015) The effect of coffee, tea, and caffeine consumption on serum uric acid and the risk of hyperuricemia in Korean Multi-Rural Communities Cohort. Rheumatol Int 35: 327–336. 10.1007/s00296-014-3061-8 24929540

[37] Yi J, Yan B, Li M, Wang Y, Zheng W, et al (2016) Caffeine may enhance orthodontic tooth movement through increasing osteoclastogenesis induced by periodontal ligament cells under compression. Arch Oral Biol 64: 51–60. 10.1016/j.archoralbio.2015.12.009 26773691

[38] Kamagata-Kiyoura Y, Ohta M, Cheuk G, Yazdani M, Saltzman MJ, et al (1999) Combined effects of caffeine and prostaglandin E2 on the proliferation of osteoblast-like cells (UMR106-01). J Periodontol 70: 283–288. 10225544 10.1902/jop.1999.70.3.283

[39] Bernabe E, Vehkalahti MM, Sheiham A, Aromaa A, Suominen AL (2014) Sugar-sweetened beverages and dental caries in adults: a 4-year prospective study. J Dent 42: 952–958. 10.1016/j.jdent.2014.04.011 24813370

[40] Koyama Y, Kuriyama S, Aida J, Sone T, Nakaya N, et al (2010) Association between green tea consumption and tooth loss: cross-sectional results from the Ohsaki Cohort 2006 Study. Prev Med 50: 173–179. 20109485 10.1016/j.ypmed.2010.01.010

[41] Markkanen H (1978) Periodontal treatment need in a Finnish industrial population. Community Dent Oral Epidemiol 6: 240–244. 281289 10.1111/j.1600-0528.1978.tb01158.x

[42] Je Y, Jeong S, Park T (2014) Coffee consumption patterns in Korean adults: the Korean National Health and Nutrition Examination Survey (2001–2011). Asia Pac J Clin Nutr 23: 691–702. 10.6133/apjcn.2014.23.4.11 25516328

[43] Lee JH, Oh MK, Lim JT, Kim HG, Lee WJ (2016) Effect of Coffee Consumption on the Progression of Type 2 Diabetes Mellitus among Prediabetic Individuals. Korean J Fam Med 37: 7–13. 10.4082/kjfm.2016.37.1.7 26885316 PMC4754290

[44] Hong JW, Noh JH, Kim DJ (2016) Factors Associated With High Sodium Intake Based on Estimated 24-Hour Urinary Sodium Excretion: The 2009–2011 Korea National Health and Nutrition Examination Survey. Medicine (Baltimore) 95: e2864. 26945369 10.1097/MD.0000000000002864 PMC4782853

[45] Chung JH, Hwang HJ, Kim SH, Kim TH (2016) Associations Between Periodontitis and Chronic Obstructive Pulmonary Disease; the 2010–2012 Korean National Health and Nutrition Examination Survey (KNHANES). J Periodontol: 1–11. 10.1902/jop.2016.150682 26912338

